# Supplementary figures and images for: Protein intake in inhabitants with regular exercise is associated with sleep quality: Results of the Shika study
Source: PLoS One. 2021 Feb 26;16(2):e0247926. doi: 10.1371/journal.pone.0247926 (PMC7909647; doi:10.1371/journal.pone.0247926)

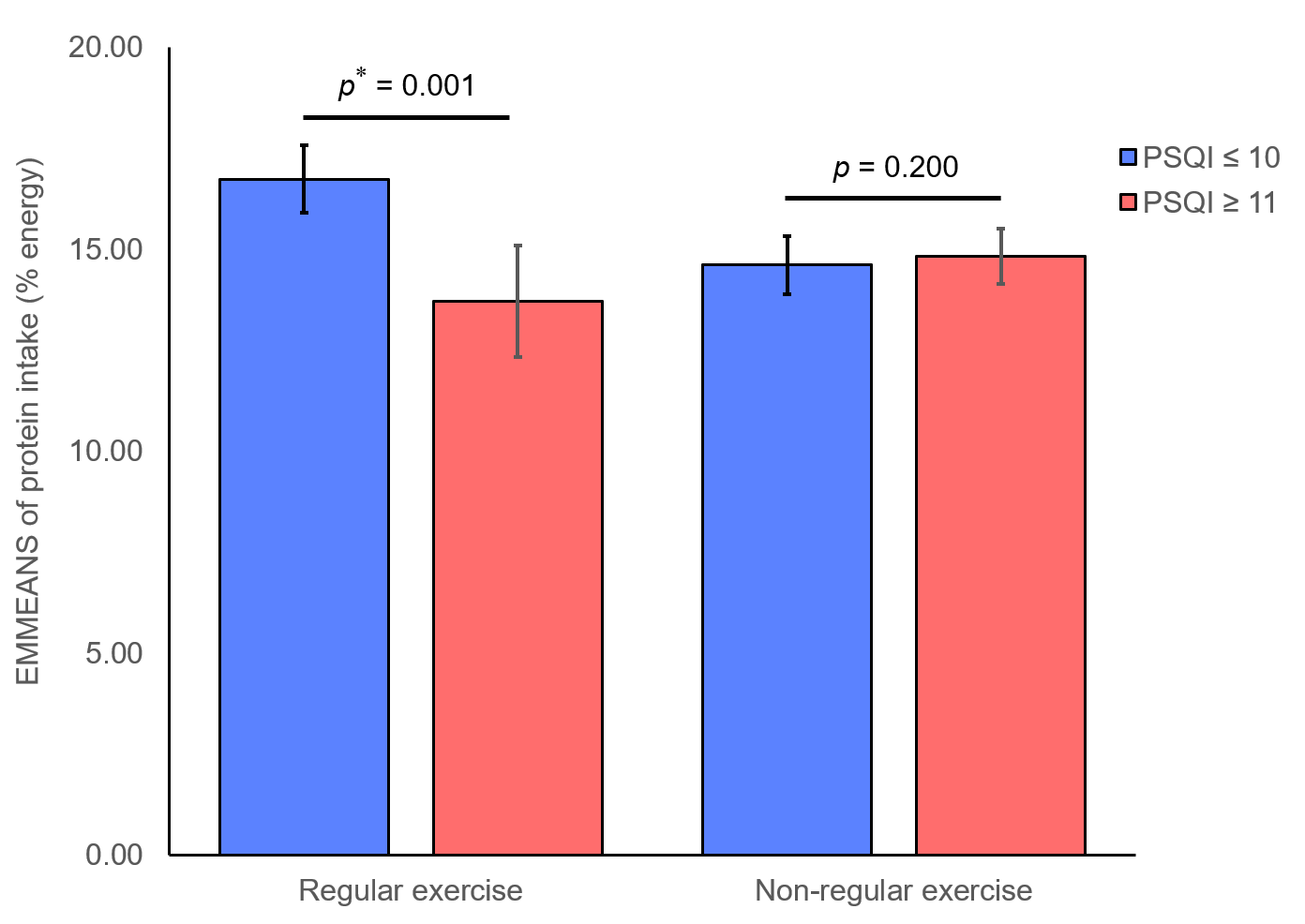

Supplement: S1 Fig — * Post hoc Bonferroni analysis. Adjusted for age = 60.54, sex = 1.51, BMI = 23.05, current smoker = 1.83, current drinker = 1.42, education = 2.32, hypertension = 1.67, diabetes = 1.92. Error bar: 95% CI. Abbreviations: PSQI, Pittsburgh Sleep Quality Index, EMMEANS, estimated marginal means. (TIF) [file pone.0247926.s001.tif]
