# Supplementary material for: Protein intake in inhabitants with regular exercise is associated with sleep quality: Results of the Shika study
Source: PLoS One. 2021 Feb 26;16(2):e0247926. doi: 10.1371/journal.pone.0247926 (PMC7909647; doi:10.1371/journal.pone.0247926)
Supplement: Attachment — Submitted filename: Response to Reviewers.docx [file pone.0247926.s002.docx]

Jun. 14^th^, 2021.

Jose M. Moran, Ph. D
Academic Editor
PLOS ONE

Dear Dr. Moran,

Thank you for inviting us to submit a revised draft of our manuscript entitled, “Protein intake in inhabitants with regular exercise is associated with sleep quality: Results of the Shika study” to PLOS ONE. We also appreciate the time and effort you and each of the reviewers have dedicated to providing insightful feedback on ways to strengthen our paper. Thus, it is with great pleasure that we resubmit our article for further consideration. We have incorporated changes that reflect the detailed suggestions you have graciously provided. We also hope that our edits and the responses we provide below satisfactorily address all the issues and concerns you and the reviewers have noted.

To facilitate your review of our revisions, the following are our point-by-point responses to the questions and comments delivered in your letter dated Nov 24th, 2020.

**Major points.**

**Q1.	Please ensure that your manuscript meets PLOS ONE's style requirements, including those for file naming.**

A1.	We have reconfirmed PLOS ONE's style requirements and corrected the file names to read Revised Manuscript and Manuscript.

**Q2.	In your Methods section, please provide additional information about the participant recruitment method and the demographic details of your participants. Please ensure you have provided sufficient details to replicate the analyses such as: a) the recruitment date range (month and year), b) a description of any inclusion/exclusion criteria that were applied to participant recruitment, c) a table of relevant demographic details, d) a statement as to whether your sample can be considered representative of a larger population, e) a description of how participants were recruited, and f) descriptions of where participants were recruited and where the research took place. Finally, please report whether sample size calculations were performed.**

A2.	We have added some descriptions to the Methods section to clarify the intended meaning. Answers to each comment are provided below:

a) The recruitment date range (month and year) has been described as follows: “In this cross-sectional study, comprehensive health survey data were collected from the residents of Shika Town, Ishikawa Prefecture, Japan, between November 2017 and February 2018” (P5 L118–120 in the original manuscript).

b) When describing the participants, the following discussion of the exclusion criteria has been added to the revised manuscript: “Of these individuals, 193 were excluded because they did not meet the survey criteria [169 participants did not complete the brief-type self-administered diet history questionnaire (BDHQ), 1 participant did not have energy records within 600–4000 Kcal/day, and 23 participants did not complete the smoking, drinking, or exercise questionnaire]” (P7 L135–139).

c) Details of the relevant demographics in Shika town have been described in the revised manuscript as follows: “As of November 2017, there were 21,007 residents in Shika Town, and 15,012 were older than 40 years [22]” (P6 L121–122).

d) We have added the following statement to the revised manuscript: “The age distribution of the participants in the present study did not differ from that of the Shika Town inhabitants” (P9 L199 –200). However, we know that there were not enough participants in this study, so we also added the following statement to the limitations section: “This study might include selection bias because the subjects participated voluntarily in this study” (P21 L397–398).

e) We have added the following phrase to the revised manuscript: “This study was conducted on participants recruited from those who underwent a medical examination in Shika Town” (P7 L132–133).

f) We noted that “a total of 378 people aged 40 years and older who live in four model districts (Horimatsu, Tsuchida, Higashimatsudo, and Togi) provided their consent to participate in this sleep study” on P6 L127–128 in the original manuscript.

Finally, since this study is epidemiologically based on the general population, we believe that the sample size is sufficient for this study, which has an epidemiologic design.

**Q3.	Please include additional information regarding the survey or questionnaire used in the study and ensure that you have provided sufficient details that others could replicate the analyses. For instance, if you developed a questionnaire as part of this study and it is not under a copyright more restrictive than CC-BY, please include a copy, in both the original language and English, as Supporting Information. Moreover, please include more details on how the questionnaire was pre-tested, and whether it was validated.**

A3.	We described the details of the questionnaire in the "Questionnaire and measurements" section (ex. items (1. yes, 2. no) on P6 L143–P7 L150 in the original manuscript. The Japanese versions of the BDHQ and PSQI were used without modifications. We have added the following phrases to the revised manuscript: “The validity of the BDHQ has been demonstrated in previous studies in Japanese populations [26, 27]” (P8 L169–170) and “The validity of the PSQI has been demonstrated in previous studies in Japanese populations [29]” (P8 L181–182).

**Q4.	We note that you have indicated that data from this study are available upon request. PLOS only allows data to be available upon request if there are legal or ethical restrictions on sharing data publicly. For information on unacceptable data access restrictions, please see http://journals.plos.org/plosone/s/data-availability#loc-unacceptable-data-access-restrictions.**

**In your revised cover letter, please address the following prompts:**

**a) If there are ethical or legal restrictions on sharing a de-identified data set, please explain them in detail (e.g., data contain potentially identifying or sensitive patient information) and who has imposed them (e.g., an ethics committee). Please also provide contact information for a data access committee, ethics committee, or other institutional body to which data requests may be sent.**

**b) If there are no restrictions, please upload the minimal anonymized data set necessary to replicate your study findings as either Supporting Information files or to a stable, public repository and provide us with the relevant URLs, DOIs, or accession numbers. Please see http://www.bmj.com/content/340/bmj.c181.long for guidelines on how to de-identify and prepare clinical data for publication. For a list of acceptable repositories, please see http://journals.plos.org/plosone/s/data-availability#loc-recommended-repositories.**

A4.	We understand the request for data disclosure; however, the disclosure of data is restricted by the Kanazawa University Ethics Committee because of the protection of personal information. The data described in the manuscript will be made available upon request for application and approval. We have added the following “non” author parson in charge information to the revised manuscript: “(Kanazawa University Ethics Committee. Parson in charge: Yuko Katsuragi < pub-jim2@staff.kanazawa-u.ac.jp >)” (P3 L51–52)

**Reviewer #1:**

**Q5.	I am struck by the division of two study groups based on a cut-off point of 10 in the PSQI without a clinical justification.
The original PSQI study concluded that a value greater than 5 indicates a negative impact on sleep. This data is mentioned by the authors in the methodology. However, it makes no mention of the Japanese validation study that found similar data. That is, values >5.5 are indicative of poor sleep quality. By the way, in the study the control group had an average 38 years old and a cut-off point of 7.5, a specificity of 97% was found for those patients with primary insomnia. Then, an average of 10 points seems to be high and difficult to be considered normal. (Doi Y, Minowa M, Uchiyama M, Okawa M, Kim K, Shibui K, et al. Psychometric assessment of subjective sleep quality using the Japanese version of the Pittsburgh Sleep Quality Index (PSQI-J) in psychiatric disordered and control subjects. Psychiatry Res. 2000;97: 165–172.)
The authors could use the cut-off points that best fit their purposes, but as a post-frame analysis. In strict adherence to the above, it would be desirable for the researchers to add an analysis considering the cut-off point of >5.5 and continue with the groups they consider.**

A5.	Since we examined the PSQI for the general population, the minimum PSQI score of the participants in this study was 6. Therefore, performing this analysis based on a cut-off point of a PSQI > 5.5 was not suitable.

**Q6.	The discussion should mention the possible causes for which the average population has poor sleep quality (mean PSQI of 10) and be careful to consider the group with <10 as having a good quality of sleep.**

A6.	We have included the following citations: “Buysse *et al*. [28] defined a PSQI score >5 as poor sleep quality. Conversely, Das *et al*. [33] demonstrated that the mean PSQI was 8.59 ± 5.35 in a community-based study among a geriatric population, and they described that the difference in the PSQI may be due to the different cultures and lifestyles of people in different countries” (P19 L337–341). We also included the following statement in the revised manuscript: “The mean PSQI of all the participants in the present study was 10.7 ± 2.7, which seemed to reflect the current average Japanese lifestyle” (P19 L341–342).

**Reviewer #2**

**Q7.	methodology:
the author needs to define what is PSQI and BDHQ and did you use Japanese version and if so, any validation study done.**

A7.	The Japanese versions of the BDHQ and PSQI were used without modifications. We have added the following phrases to the revised manuscript: “The validity of the BDHQ has been demonstrated in previous studies in Japanese populations [26, 27]” (P8 L169–170) and “The validity of the PSQI has been demonstrated in previous studies in Japanese populations [29]” (P8 L181–182).

**Q8.	Discussion:
PSQI has its own drawback for sleep quality assessment. furthermore, people with metabolic syndrome are at greater risk of developing obstructive sleep apnea and that may hinder their sleep quality
please add these comments in the discussion.**

A8.	We have added the following limitation to the revised manuscript: “Since the PSQI was evaluated only with a questionnaire, using a more objective method such as a polysomnogram to assess sleep quality is necessary” (P21 L398–399). Our results (Table 4) indicated that metabolic syndrome had no major effect on the PSQI and that there was no interaction between the PSQI and regular exercise. Therefore, we believe that the effects of metabolic syndrome on the results of the present study were not significant. However, we have added the following statement to the revised manuscript: “Since we have not examined the effects of obstructive sleep apnea, future studies should be performed that incorporate a design to evaluate obstructive sleep apnea” (P21 L399–401).

**Other**

**Q9.	Do you want your identity to be public for this peer review? For information about this choice, including consent withdrawal, please see our Privacy Policy.**

**Reviewer #1: No**

**Reviewer #2: No**

A9.	I want my identity to be published for this peer review.

Again, thank you for giving us the opportunity to strengthen our manuscript with your valuable comments and queries. We have worked hard to incorporate your feedback and hope that these revisions persuade you to accept our submission.

Sincerely,

Fumihiko Suzuki

Department of Environmental and Preventive Medicine, Graduate School of Medical Science, Kanazawa University, 13-1 Takaramachi, Kanazawa, Ishikawa 920-8640, Japan.

Tel: +81-76-265-2218

Email address: [f-suzuki@stu.kanazawa-u.ac.jp](mailto:f-suzuki@stu.kanazawa-u.ac.jp)
