# Supplementary material for: Protein intake in inhabitants with regular exercise is associated with sleep quality: Results of the Shika study
Source: PLoS One. 2021 Feb 26;16(2):e0247926. doi: 10.1371/journal.pone.0247926 (PMC7909647; doi:10.1371/journal.pone.0247926)
Supplement: Attachment — Submitted filename: Response to Reviewers.docx [file pone.0247926.s003.docx]

Feb 16^th^, 2021.

Jose M. Moran, Ph. D
Academic Editor
PLOS ONE

Dear Dr. Moran,

Thank you for inviting us to submit a revised draft of our manuscript entitled, “Protein intake in inhabitants with regular exercise is associated with sleep quality: Results of the Shika study” to PLOS ONE. We also appreciate the time and effort you and each of the reviewers have dedicated to providing insightful feedback on ways to strengthen our paper. Thus, it is with great pleasure that we resubmit our article for further consideration. We have incorporated changes that reflect the detailed suggestions you have graciously provided. We also hope that our edits and the responses we provide below satisfactorily address all the issues and concerns you and the reviewers have noted.

To facilitate your review of our revisions, the following are our point-by-point responses to the questions and comments delivered in your letter dated Feb 11th, 2020.

**Major points.**

**Q1.	Although authors have appropriately addressed the requirements of the reviewers, before recommending publication of the article, and given that the authors have included in their analysis the t-Student, ANCOVA, multiple linear regression and Pearson correlations, the authors should state in the methodology section that all the variables involved fulfilled the assumptions required by parametric methods and that these were tested by the appropriate tests (indicate which ones the authors have used).**

A1.	We have added the following phrase to the revised manuscript: “The distribution of variables was checked by the Kolmogorov–Smirnov, and Shapiro–Wilk normality tests, or the normal distribution curve in the histogram was confirmed before using other statistical tests.” (P9 L180–182).
Supplementary explanation: Although some variables had a *p*-value of 0.05 or less in the Kolmogorov–Smirnov, and Shapiro–Wilk normality test, it was confirmed that a normal distribution curve was drawn in the histogram. Therefore, we believe that the variables used in this study can be expected to have a multivariate normal distribution.

**Reviewer #1:**

**There were no additional comments.**

**Reviewer #2**

**Q1.	Has the statistical analysis been performed appropriately and rigorously? (Reviewer #2: I Don't Know)**

A1.	We have added the following phrase to the revised manuscript: “The distribution of variables was checked by the Kolmogorov–Smirnov, and Shapiro–Wilk normality tests, or the normal distribution curve in the histogram was confirmed before using other statistical tests.” (P9 L180–182).
Supplementary explanation: Although some variables had a *p*-value of 0.05 or less in the Kolmogorov–Smirnov, and Shapiro–Wilk normality test, it was confirmed that a normal distribution curve was drawn in the histogram. Therefore, we believe that the variables used in this study can be expected to have a multivariate normal distribution.

**Q2.	minor linguistic errors,**

**i would suggest writing a review linking, sleep, exercise fitness and diet. the focus on sleep not only from OSA prospective but rather descriping the link between sleep duration, timing and physical fitness in addition to healthy diet.**

A2.	Since we were conducting a cross-sectional analysis, we cannot examine the sleep duration and timing in detail, but the mechanism by which regular exercise and nutrition on sleep quality were described as follows: "The reason for good sleep quality among participants who reported high protein intake in their daily diet and regular exercise was thought to be a result of the pathogenesis in which the tryptophan-serotonin-melatonin pathway was activated due to the enhanced protein absorption" (P20 L348-351).

Again, thank you for giving us the opportunity to strengthen our manuscript with your valuable comments and queries. We have worked hard to incorporate your feedback and hope that these revisions persuade you to accept our submission.

Sincerely,

Fumihiko Suzuki

Department of Environmental and Preventive Medicine, Graduate School of Medical Science, Kanazawa University, 13-1 Takaramachi, Kanazawa, Ishikawa 920-8640, Japan.

Tel: +81-76-265-2218

Email address: [f-suzuki@stu.kanazawa-u.ac.jp](mailto:f-suzuki@stu.kanazawa-u.ac.jp)
